# Consensus statement on enzyme replacement therapy for mucopolysaccharidosis IVA in Central and South-Eastern European countries

**DOI:** 10.1186/s13023-022-02332-7

**Published:** 2022-05-10

**Authors:** Martin Magner, Zsuzsanna Almássy, Zoran Gucev, Beata Kieć-Wilk, Vasilica Plaiasu, Anna Tylki-Szymańska, Dimitrios Zafeiriou, Ioannis Zaganas, Christina Lampe

**Affiliations:** 1 Department of Paediatrics and Inherited Metabolic Disorders, General University Hospital and First Faculty of Medicine, Charles University, KPDPM 1. LF UK a VFN v Praze, Ke Karlovu 2, 128 08 Prague, Czech Republic; 2 Department of Toxicology and Metabolic Diseases, Heim Pal National Pediatric Institute, Budapest, Hungary; 3 University Children’s Hospital, Skopje, North Macedonia; 4 Unit of Rare Metabolic Diseases, Department of Metabolic Diseases, Jagiellonian University Medical College, University Hospital, Krakow, Poland; 5 Regional Centre of Medical Genetics, INSMC Alessandrescu-Rusescu, Bucharest, Romania; 6 Department of Pediatric Nutrition and Metabolic Diseases, The Children’s Memorial Health Institute, Warsaw, Poland; 7 First Department of Pediatrics, Hippokratio General Hospital, Aristotle University, Thessaloniki, Greece; 8 Neurogenetics Laboratory, Neurology Department, University Hospital of Heraklion, University of Crete, Heraklion, Greece; 9 Department of Child Neurology, Epileptology and Social Pediatrics, Centre for Rare Diseases, University of Giessen, Giessen, Germany

**Keywords:** Morquio syndrome A, Mucopolysaccharidosis IVA, Central and South-Eastern European countries, Enzyme replacement therapy, Elosulfase alfa

## Abstract

**Background:**

Mucopolysaccharidosis IVA (MPS IVA), or Morquio A syndrome, is a rare inherited metabolic disorder caused by deficiency of the lysosomal enzyme N-acetylgalactosamine-6-sulfatase. A progressive systemic skeletal chondrodysplasia, leading to significant morbidity and reduced life expectancy is the main clinical feature of this multisystemic disease. Although enzyme replacement therapy with elosulfase alfa is established in Europe, the rarity of disease and other factors still set hurdles in having patients treated in some countries. Aim of this statement is to provide evidence-based guidance for the enzyme replacement treatment of Morquio A patients, harmonizing recommendations from published guidelines with the real-life clinical practice in the Central and South-Eastern European region.

**Participants:**

The Consensus Group, convened by 8 Steering Committee (SC) members from 7 Central and South-Eastern European countries, consisted of a multidisciplinary group of 17 experts in the management of MPS in Central and South-Eastern Europe.

**Consensus process:**

The SC met in a first virtual meeting with an external scientific coordinator, to discuss on clinical issues to be analyzed in guidance statements. Statements were developed by the scientific coordinator, evaluated by the SC members in a first modified-Delphi voting and adapted accordingly, to be submitted to the widest audience in the Consensus Conference. Following discussion and further modifications, all participants contributed to a second round of modified-Delphi voting.

**Results:**

Nine of ten statements, concerning general guidelines for management of MPS IVA patients and specific recommendations for treatment, received final consensus.

**Conclusions:**

European guidelines and evidence-based recommendations for Morquio A patients should be considered in the real life of Central and South-Eastern European countries and adapted to unique clinical practice approaches and criteria for patients’ access to treatment and reimbursement in the region.

## Background

Mucopolysaccharidosis IVA, or Morquio A syndrome, is caused by defects in the enzyme N-acetylgalactosamine-6-sulfate sulfatase (GALNS), leading to abnormal accumulation of keratansulfate (KS) and chondroitin-6-sulfate (C6S) in multiple tissues, mainly bone, cartilage, heart valves, and cornea [1]. Clinically, MPS IVA causes systemic skeletal spondyloepiphyseal dysplasia, through excessive storage of KS undegraded products in the cartilage. Peculiar clinical features derive from the disruption of cartilage, probably due to abnormal chondrogenesis and/or endochondral ossification and typically include a marked short stature, odontoid hypoplasia, protrusion of the chest, kyphoscoliosis, platyspondyly, *coxa valga*, abnormal gait, and laxity of joints. These clinical features, with preservation of intelligence, distinguish Morquio A from other types of MPS [2]. Other potential complications include pulmonary impairment, valvular heart disease, hearing loss, hepatomegaly, fine corneal clouding, coarse facial features, and widely spaced teeth with abnormally thin enamel and frequent caries [3]. Due to allelic heterogeneities in the GALNS gene, MPS IVA manifests across a broad spectrum of phenotypes, ranging from severe or ‘classical’ forms, characterized by early clinical manifestations (before 1 year of age) and rapid progression of musculoskeletal symptoms, to a later onset and slowly progressing ‘mild’ disease, characterized by less significant bone involvement (‘non-classic’ disease) [4]. An intermediate form is also described, generally diagnosed between 1–5 years old. The severity of symptoms is determined by the degree of skeletal and joints’ deterioration. Classical forms are generally prevalent, affecting almost 70% of individuals in the International Morquio Registry [5]. Although patients with MPS IVA generally appear healthy at birth, soon skeletal deformities become evident and require intervention within a few years of age [3]. Spinal cord compression is a significant cause of morbidity for MPS IVA patients [6], thus many patients undergo surgical interventions in the upper cervical spine until they reach a mean age of 10 years [5]. Lower extremities surgery is also recommended in most patients, to reduce *coxa valga,* and *genu valgum*, otherwise causing debilitating pain and early loss of ambulation [7]. Other lower extremity impairments, such as knee flexion and external tibial torsion, require orthopedic surgeries to improve function and quality of life. Knee deformity, for instance, is the most common feature of MPS IVA in children, commonly observed at around 3 years of age [8]. Although surgical treatment may improve skeletal abnormalities, it generally fails to provide long-lasting results and patients often become severely handicapped and wheelchair bound when they are teenagers. Average life expectancy is 20–30 years for patients with classical disease, who usually die from respiratory problems, cervical spinal cord complications or heart valve disease, while individuals with mild forms may survive through their 60 s and 70 s [5].

At present, the only etiologic treatment for Morquio A syndrome with efficacy validated in clinical studies is enzyme replacement therapy (ERT) [9–11]. Approved by the FDA and EMA for the use in children and adults with Morquio A syndrome in 2014, ERT for MPS IVA is based on treatment with recombinant human GALNS, or elosulfase alfa, for intravenous administration [12]. In clinical studies, elosulfase alfa treatment led to sustained reduction of urinary KS excretion and improvement and/or stabilization in endurance, measured by the six-minutes walk test (6MWT) and the three-minutes stair climb test (3MSCT), with a good tolerance profile [13]. Positive effects of elosulfase alfa treatment are evident if compared to natural history of disease [14–16]. The slower progression of disease in patient treated with elosulfase alfa also reflected in improved pulmonary function and increased patients’ ability to perform activities of daily living (ADL), lessening their need for caregiver assistance. Other patients’ reported outcomes (PRO), such as pain severity and quality of life (QoL), measured as the EQ-5D-5L utility score (European Quality of Life—five dimensions-five levels questionnaire—mobility, self-care, usual activities, pain & discomfort, anxiety & depression), significantly improved in treated patients of different ages [13].

Although the European guidelines for management of MPS IVA have been published [17], their practical application might be challenging. In fact, several gaps in treatment accessibility recently emerged in "The Mucopolysaccharidosis Management Physician Survey", performed in 14 Southern-Eastern European countries (Bulgaria, Croatia, Cyprus, Czech Republic, Greece, Hungary, Lithuania, Montenegro, Poland, North Macedonia, Romania, Serbia, Slovakia, Slovenia) [18]. Specifically, elosulfase alfa is not available for patients of all ages in all countries included in the study and it is not accessible at all in 12.5% of 16 reference centers analyzed. Also, reimbursement is a main hurdle to treatment, as it relies on individual patient request to health insurance authorities in 70% of reference centers. Thus, the survey revealed the need for facilitation and extension of ERT for MPS IVA to more patients. Significantly, elosulfase alfa use in adult patients is limited compared to children (21% of adult patients are on ERT *vs*. 56% of children). Also, concerning the evaluation of important outcomes for monitoring response to ERT in MPS IVA patients, several inconsistencies with current European guidelines emerged in some countries, due to organizational reasons or availability of examinations. For instance, respiratory function is difficult to perform in almost one third of centers. Similarly, surgery, anesthesiology and sleep studies, although considered important outcomes for monitoring ERT efficacy by most participants, are difficult to perform in many centers. Also, frequency of follow-up examinations is not standardized at a regional level and, most importantly, only a minority of experts declared to have a clear definition of satisfactory (or non-satisfactory) response to ERT [18].

In this context, with this work we aimed to reach a consensus among expert physicians in Central and South-Eastern European countries on initiation and follow-up of elosulfase alfa treatment in patients with MPS IVA and provide a shared set of evidence-based guidance, with the aim of harmonizing recommendations from current guidelines and long term studies with real-life clinical practice in the region.

## Methods

### Development of guidance statements

This consensus process is the natural continuation of the multinational expert physicians’ survey analysis on the state of art of MPS IVA management and treatment in Central and South-Eastern European countries, conducted between March 2020 and May 2021, that revealed the need of improving overall disease management and treatment accessibility in the region. A group of 8 physicians from 8 reference centers in 7 different countries, with specific expertise in MPS care, was identified as the Steering Committee to guide the consensus process. All members of the SC were also involved in the previous multinational survey analysis, to maintain continuity of the process. The SC met in a first Virtual Working Group Meeting with the scientific coordinator of the program, where they identified a list of clinical topics to be addressed in the statement. During the workshop, the SC shared experiences and knowledge on the topics, addressing unique features of clinical practice in different countries, in order to reach an agreement on interventions needed to optimize clinical approaches for MPS IVA management and pharmacotherapy at a regional level. Based on the discussion during the first Working Group Meeting, guidance statements were developed by the scientific coordinator and sent to all members of the SC for the first round of modified-Delphi voting. The SC then met with the scientific coordinator in a second virtual Working Group Meeting to refine the guidance statements to be submitted for a second modified-Delphi Consensus in a wider audience of experts. The scientific coordinator led and supervised the whole process of consensus, although maintaining neutrality and not participating to voting.

The program was sponsored by BioMarin Pharmaceuticals. All the meetings and the SurveyMonkey voting were organized by CD Pharma, an independent consulting company, with no intervention of the sponsor in statement development or discussion.

### Consensus process

Guidance statements were first sent to all members of the SC for consensus evaluation in a modified-Delphi survey, using the SurveyMonkey platform (https://it.surveymonkey.com). Participants expressed their level of agreement/disagreement on each statement anonymously, using a 4‐point Likert‐type scale (1 = strongly disagree, 2 = somewhat disagree, 3 = somewhat agree, 4 = strongly agree). The number and percentage of participants who scored each item as 1 or 2 (disagreement), and as 3 or 4 (agreement) were calculated. Consensus was defined as a sum for agreement ≥ 66%. All statements that achieved < 66% of full agreement in this first voting were further discussed by the SC during the second virtual Working Group Meeting, refined and rephrased for clarification, and all statements were then presented to all participants for further discussion in the Consensus Conference. During the conference statements were discussed by all participants and further modified. According to the Delphi methodology, the statements were submitted to all participants for immediate voting, using a 2‐point Likert‐type scale (1 = disagree and 2 = agree). In this round, consensus was defined as a sum for agreement ≥ 75%.

## Results

A total of 11 guidance statements were initially developed by the scientific coordinator and analyzed within the SC, based on discussion on long term studies and current guidelines on MPS IVA management and treatment. Statements were defined as “General statements”, concerning recommendations for the initiation of ERT in MPS IVA patients and baseline assessments to perform, and “Specific statements”, related to continuation of treatment, follow-up assessments for validation and eventual discontinuation based on patients’ characteristics, disease related events and tolerability. After first Delphi voting among the SC, 10 statements reached large consensus (≥ 83%) and 1 statement achieved 66% of agreement. Statements were slightly modified and rephrased for clarification and then discussed in a wider audience of experts in the consensus conference. The discussion led to reformulation and merge of some statements, with 9 out of 10 statements receiving final consensus. Results presented below include the final consensus statements and an outline of the rationale behind them.

### General statements

ERT with elosulfase alfa is currently the only disease modifying intervention approved for treatment of patients with MPS IVA. Efficacy of elosulfase alfa on natural history of disease has been widely demonstrated in clinical studies. Importantly, elosulfase alfa treatment is able to temporarily reestablish GALNS function and increase the degradation of accumulated KS and C6S that cause the Morquio A clinical symptoms. Accordingly, elosulfase alfa treatment results in improvement of disease related clinical outcomes, as demonstrated by ameliorated endurance, a primary efficacy outcome, measured as the 6MWT at 12 and 24 weeks [9]. Considering best evidence of positive effects of elosulfase alfa on clinical and PRO measures, treatment is recommended in the last European Guidelines.

Data on benefits of early introduction of elosulfase alfa on disease burden are limited so far; however, considering results of clinical studies, it is reasonable to assume that initiation of treatment as early as possible after diagnosis would provide positive effects on the natural history of disease [17].

Importantly, initiation of treatment should be evaluated on an individual basis, considering benefits in relation to disease burden for each patient. Thus, a comprehensive evaluation of the patient is recommended, considering disease burden, comorbidities and prognosis, also bearing in mind that benefits of treatment may not be comparable across all patients. In fact, the risk–benefit ratio and the efficacy versus cost effectiveness may be uncertain, particularly in patients with less severe disease [17, 19].

Concerning baseline and follow-up assessments to perform during treatment, guidelines are based on evidence and recommend baseline and regular follow-up of main clinical outcomes related to disease progression [17]. “The Mucopolysaccharidosis Management Physician Survey" also evaluated the real life of clinical evaluations performed in Southern and Eastern European countries in MPS IVA patients, comparing clinical and PRO measures included in European guidelines with assessments considered appropriated by the multidisciplinary expert board to evaluate treatment outcomes. Most expert physicians reported that endurance, as the 6MWT, and respiratory function examination, using the FVC or FEV1, are considered adequate assessments to evaluate ERT efficacy [18].

Importantly, longitudinal studies also showed that both clinical and patient reported outcomes in response to elosulfase alfa treatment are not influenced by pathogenic variants. Both endurance (6MWT), respiratory function (as FEV1/FVC, Forced Expiratory Volume in 1 s / Force Vital Capacity) as well as ADLs remained stable or improved in patients treated with elosulfase alfa, regardless of causative pathogenic variant(s) in a population of patients in Quebec (Canada), over 12 months. The mean improvement from baseline in the 6MWT was 23% and most patients improved in at least one MPS-HAQ domain. Endurance and ADL generally continued to improve or maintained stable in the long-term follow-up (up to 7 years) [20]. Increasing cardiac impairment with aging was also described in the MorCAP study, with cardiac valve regurgitation commonly seen in echocardiograms—tricuspid regurgitation in 34% of patients, mitral regurgitation in 25%, aortic regurgitation in 19%, and pulmonary regurgitation in 14% of subjects. Importantly, while aortic valve stenosis was relatively infrequent in the younger study population, incidence increased with age, with 16% of adult patients presenting stenosis, compared to only 4% of patients ≤ 18 years of age [14]. Progression of cardiac disease with aging was also observed in 4 of 19 patients in a longitudinal observational study [20]. Thus, rationale and evidence-based recommendation of cardiac function follow-up evaluation is included in the European guidelines [17] and it was also considered appropriate by experts participating in “The Mucopolysaccharidosis Management Physician Survey" [18].

Also, stabilization in patient reported pain severity by the BPI Short Form (BPI-SF), a standard tool to measure pain severity, pain location and the impact of pain on daily functioning, were observed in clinical studies at 1 year [21]. Due to overall detriment of QoL, patients with mucopolysaccharidoses may also experience social isolation and low self-esteem, leading to behavioral and mental health conditions such as anxiety and depression [22]. Treatment with elosulfase alfa led to stabilization of the standard Beck Depression Inventory (BDI) score [23] below the threshold of concern, and scores remained stable during long term follow-up [13]. Evaluation of disease burden indexes (PROs, EQ-5D-5L, MPS-HAQ) was also defined important for treatment monitoring by the large majority of experts in “The Mucopolysaccharidosis Management Physician Survey" [18], according to published guidelines and observational, clinical and extension studies [9, 10, 13, 17, 20, 24] (Table 1).

**Table 1:** General statements

	Consensus %
**Statement 1** ERT with elosulfase alfa should be available for all MPS IVA patients independent of age and genetic variant	100
**Statement 2** Treatment with elosulfase alfa should be initiated as early as possible	100
**Statement 3** Baseline assessments as well as regular follow-up assessments should be performed as described in the guidelines: 1. 6MWT or another appropriate test 2. uKS levels 3. pulmonary function (FVC, FEV1) 4. cardiac function (LVEF) 5. PROs QoL: MPS-HAQ Caregivers Domain, EQ-5D-5L Pain: Brief Pain Inventory (BPI) Depression: Beck Depression Inventory (BDI)	100

### Specific statements

Considering cost/effectiveness and the risk/benefits ratio of ERT, an accurate definition of appropriate parameters and time intervals to assess therapy efficacy and safety is of the outmost importance. Εvaluation of efficacy is also related to the necessity of producing evidence of effectiveness in some countries, to obtain renewal of treatment authorization or reimbursement.

Current European guidelines recommend evaluating continuation of treatment on an individual patient basis, with discontinuation criteria assessing the burden of infusion versus benefits of treatment [17]. In fact, infusion may represent an unbearable burden for some patients, who may not be compliant with the treatment schedule. In long term clinical studies, patients who underwent orthopedic surgical procedures or were non-compliant with the study protocol were excluded from the study population. Non-compliance was defined as missing ≥ 20% of ERT infusions [24]. Excluding patients who underwent surgery is understandable in clinical studies, but not for defining non-compliance in this consensus statement. Specifically, reasons for discontinuation of treatment not deriving from patients’ will (surgeries or other severe medical conditions, problems in drug delivery or organizational issues of the hospital) were excluded from the definition of non-compliance. On the other hand, continuation of treatment has to be accompanied by regular follow-up [17] and only patients who adhere regularly to follow-up assessments should be maintained on treatment. Also, the possibility of immune-related severe hypersensivity reactions has to be considered [16, 25]. Although most anaphylaxis events observed in clinical studies were mild to moderate in severity [9, 11, 16, 24], the eventuality of severe-life threatening AEs should be taken into account. Thus, according to the Risk Management Plan accompanying elosulfase alfa, ERT must be administered with the appropriate medical support readily available and infusion must be immediately stopped and emergency measures undertaken if severe AEs occur [25].

According to the group’s consensus, particular consideration about initiation or continuation of treatment should be dedicated to patients with severe phenotypes, at a very late stage of disease. In these patients, evaluation of potential benefits of ERT (possibility to improve or stabilize a severely compromised clinical condition) should be based on an accurate consideration of the patient’s disease state, also weighing the infusion burden and risk/benefit ratio [17]. This is particularly relevant for adult patients, who often present for ERT with significant disease burden. Accordingly, an individualized patient approach was considered appropriate for all patients on elosulfase alfa treatment. Specifically, discontinuation of treatment should be considered if clinical improvement is not observed after 3 years, as described in guidelines [17] and recommended in statement 4. Decision for treatment discontinuation should be taken by the physician and the patient, based on the clinical condition and/or patient’s will to continue treatment.

Long term evidence of elosulfase alfa effectiveness in reducing or stabilizing clinical and patient reported outcomes in Morquio A patients also provide rationale for long term follow-up and continuation of treatment. In the MAA study [13], real world data showed that mean uKS levels rapidly diminished by 19.13–20.75% from baseline in patients treated with elosulfase alfa and remained stable for at least 10 years. Endurance also improved, with an initial increase in 6MWT—mean 6MWT distance, 217.05 m at baseline, increased to 243.92 m at last follow-up—followed by a stabilization observed for at least 4.9 years. A 10% increase from baseline in the 6MWT (or in the timed 25-foot (7.6 m) walk, T25FW, a comparable score that can be measured also on wheelchair bound patients) was the minimum requirement for treatment continuation. Pulmonary function was also stable or improved over time, regardless of age at treatment initiation, with a mean percent change in FVC from baseline to the last measurement of 16.14% at 5.5 years; mean FEV1 also improved by 15.59% from baseline over the same period. Improvement in FVC or FEV1 of ≥ 5% over baseline, or stabilization after 1 year, was the criterion for continuation of ERT in this study. Cardiac function, as the left ventricular ejection fraction (LVEF) was also evaluated at baseline and last follow-up; a slight improvement in mean LVEF was observed and all patients had a LVEF within the normal range at last follow-up. Importantly, a decrease of less than 10% in the LVEF was considered as an inclusion criterion for maintenance in treatment. PRO criteria evaluated for follow-up were the EQ-5D-5L score or the MPS-HAQ Caregiver Burden score, the Beck Depression Score and the APPT/BPI pain severity score (depending on age). No adverse change in two out of these three scores was the minimum criterion for continuation of treatment defined in the MAA study. According to the group’s consensus, improvement in one of the three PRO scores was considered a sufficient condition for maintaining a patient on treatment, considering that disease phenotypes widely vary and improvements might be less evident in more attenuated forms. Accordingly, improvement in at least one clinical and one PR outcomes were considered appropriate in consensus for validation of treatment. Validation of treatment is also a main criterion for decision of the treating physician on ERT continuation. Still, considering that improvement or stabilization of symptoms, as defined above, could not be consistent in patients with different disease severity, a timeframe of 3 years was considered reasonable, to maintain on treatment also those patients showing slower improvement. If the above-mentioned criteria of improvement are not met, the stabilization of disease or a significant deceleration of its progression should be taken into account while validating the treatment (Table 2).

**Table 2:** Specific statements

	Consensus %
**Statement 4** To continue treatment, patients must show improvements or stabilization of disease as measured by clinical scales, laboratory markers, and patient-reported outcomes Validation of treatment should be shown in at least 1 clinical and 1 patient-reported outcome (a and b): a) clinical Improvement in 6MWT distance or the timed 25-foot (7.6 m) walk (T25FW) of ≥ 10% over baseline or stabilization after 10% improvement Any improvement in FVC or FEV1 over baseline or stabilization after 1 year Decline in LVEF < 10% from baseline or stabilization after 1 year Decline of uKS of ≥ 20% from baseline b) PROs no adverse change in numerical value of 1 out of 3 of the following: Eq-5D-5L OR MPS-HAQ Beck Depression Score (≥ 13 yrs) BPI pain severity score	100
**Statement 5** Due to the clearly defined baseline, follow-up assessments and the definition of “stabilization and improvement” (see statement 4), all adult patients with MPS IVA should have early access to treatment with elosulfase alfa	18 (this statement was not approved as it was considered redundant)
**Statement 6** In patients with MPS IVA in a very advanced stage of disease, treatment of elosulfase alfa must be discussed individually	100
**Statement 7** Treatment with elosulfase alfa in MPS IVA patients could be stopped if stabilization or improvement (as described in statement 4) is not fulfilled after 3 years of treatment or upon physician or patient decision	94
**Statement 8** Treatment with elosulfase alfa in MPS IVA patients should be stopped if severe unmanageable infusion related reactions or an additional life-threatening disease occur	100
**Statement 9** Treatment with elosulfase alfa in MPS IVA patients could be stopped if the patient is not compliant regarding follow-up assessments	100
**Statement 10** Treatment with elosulfase alfa in MPS IVA patients could be stopped if the patient missed ≥ 20% of their scheduled elosulfase alfa infusions, excluding surgeries or other severe medical conditions, problems in drug delivery or organizational issues of the hospital	100

## Discussion

MPS IVA is a severe and progressive disease causing extensive morbidity and early mortality, with an estimated prevalence ranging from 1 in 76,000 to 1 in 640,000 live births [26]. As for all rare diseases, despite established clinical guidelines, Morquio A still represents a significant unmet medical need, mainly due to poor awareness of disease management and specific patients’ needs in the health care systems. Particularly, with progress in care and implementation of ERT in last years, age of patients with Morquio A significantly increased, thus the issue of transition of patients to adult care became clinically relevant and novel arguments on management of these patients emerged [27, 28]. “The Mucopolysaccharidosis Management Physician Survey" recently described the real life of MPS IVA management in Central and South-Eastern Europe, outlining a positive picture for disease management in the region, while revealing critical hurdles to ERT accessibility, particularly for adult patients, as well as several inconsistencies in follow-up implementation [18]. In this context, this consensus statement aims to provide a set of clinical guidelines for treatment and follow-up of Morquio A patients in Central and South-Eastern European countries, to optimize clinical practice, align to widely shared recommendations and improve care.

The discussion was a main opportunity for sharing different positions coming from main reference centers for MPS in different countries, comparing real life management and analyzing pros and cons of diverse clinical practice approaches. As a result, the consensus process had a highly informative role, providing a wide audience with the opportunity of considering best clinical practices not fully implemented in all countries and evaluate them in real-life settings. All guidance statements were initially developed by the scientific coordinator and then rephrased and fine-tuned exclusively by the SC, based on evidence, expert opinion and published guidelines, without any influence from the sponsor. Moreover, the whole consensus process was managed by an independent communication agency, to maintain editorial independence from the sponsor. Experts were enrolled based on their expertise in management of Morquio A. Particularly, as Morquio A is a complex multisystemic disease, physicians with diverse medical education were invited for modified-Delphi voting. Thus, our methodology provides reliability and strength to the consensus process, due to the diverse medical background and geographic localization of the SC members and expert audience. The anonymous voting method confers credibility to the surveys and ensures that voting decisions were taken independently, without biases, in accordance with the Delphi methodology [29]. Further validating the methodology for consensus, all statements proposed by the scientific coordinator reached consensus after a first voting round within the SC, confirming the adequacy of clinical topics discussed.

Importantly, this consensus statement derives from merging best evidence, clinical guidelines and the real life clinical practice in the Central and South-Eastern European region. Discussion mainly focused on region-specific unmet medical needs, the availability of treatment and follow-up assessments in different countries and the necessity to provide validation of treatment efficacy for renewal of authorization by the Health Authorities in some countries. Actually, as emerged in "The Mucopolysaccharidosis Management Physician Survey" [18], ERT for MPS IVA patients is not available for all patients in all centers, due to reimbursement and regulatory barriers. Hurdles particularly limit treatment of adult patients. Moreover, criteria for follow-up of MPS IVA patients are not evenly implemented at a regional level. With this consensus statement we aim to improve awareness on the importance of ERT availability for all patients, in order to standardize practice and improve care. In this framework, guidance statements were developed in full accordance with published guidelines, although avoiding excessively strict recommendations about time for follow-up and criteria for patient assessment and validation of treatment, to leave an acceptable decision frame for physicians, considering different rules and schedules for patient assessment in different countries. Accordingly, the use of “should” and “must” in statements identifies more stringent guidance, where decision about treatment or follow-up has to be unarguably bound to clinical recommendations. It is the case of the strong recommendation of availability of ERT for all patients, where rationale is supported by evidence-based efficacy of elosulfase alfa in modifying natural history of disease in patients of different age, regardless of genetic variants [13, 20]. At this regard, two different statements were approved at first voting round, one recommending treatment irrespective of age and the second regardless of causative variant(s). During the second consensus conference in the widest audience, these two statements were combined, since consideration of both criteria together was deemed more appropriated. This statement is particularly relevant in those countries where treatment is not available for all patients, as revealed by “The Mucopolysaccharidosis Physician Survey” [18].

“Should” was also used in recommendations for follow-up and validation of treatment. Nevertheless, although follow-up schedules must be compliant with published guidelines [17] and real-life evidence from the MAA study [13], the schedule for follow-up was thoroughly discussed. Final statements (3 and 4) were reformulated to harmonize them to particular requirements for patient management and follow-up in different countries and to real life availability of assessments, while leaving a reasonable margin for decision to the treating physician, according to real life care conditions and individual patient needs. Accordingly, no precise timeline for follow-up was included, rather recommending performing “regular follow-up” as described in published guidelines (with European guidelines recommending “regular” follow-up every year) [17]. Concerning the inclusion criteria for continuation of treatment (statement 4), stabilization is regarded as an absence of disease progression or prevention of the significant progressive worsening of clinical and PRO parameters observed in the natural course of disease. “Improvement or stabilization” mentioned in statement 4 should be considered as a sufficient criterion for validation of treatment [14, 15]. Improvement in endurance should not be evaluated exclusively through the 6MWT but an additional appropriate test is also recommended, suggesting the T25FW, as it is not based exclusively on walking, so that patients, particularly if wheelchair bound, can perform it in other ways (i.e. rolling, crawling). Also, “any improvement” in pulmonary function was decided to be more suitable during second statement discussion and voting, compared to the initial statement “improvement in FVC or FEV1 of ≥ 5% over baseline”. The initial statement, implemented in the MAA study [13], was considered limiting, as excluding patients with less severe disease, often showing a smaller improvement at follow-up. Importantly, guidelines in the MAA study suggest that 4 out of 5 parameters, among clinical and PRO measures, should be fulfilled to maintain treatment. Initially, statement 4 was formulated identical to this recommendation. However, in the first consensus conference within the SC, it was reformulated, and “at least 1 clinical and 1 patient-reported outcomes” was judged more appropriate and reasonable. The latter statement considers established hospital protocols, availability of assessment methods, the largely variable natural history of disease and the need for more patients to benefit from ERT. Maintenance of treatment will be reconsidered if stabilization or improvement is not obtained after 3 years of treatment or upon physician or patient decision. A 3-year period was considered more adequate than 1 year (as in the MAA study), as it leaves more time to observe progression in patients with more attenuated disease, and also represents a more reasonable timeframe for presenting evidence of efficacy to the Health Authorities for renewal of authorization.

Together with the criteria for treatment maintenance and follow-up, the concept of “improvement or stabilization” was defined, representing the rationale for recommendation of early access of all patients to ERT. Initially, statement 5 received consensus within the SC, then it was decided not to approve it in the second consensus conference, since it was considered merely redundant after discussion and reformulation of other statements.

In any case, the need of offering elosulfase alfa treatment to all patients was reaffirmed, regardless of age or pathogenic variants, also including patients at an advanced stage of disease. Reflecting the approach behind this consensus, decision about treatment in these patients is left to the treating physician, after evaluation of the cost/effectiveness and risk/benefit ratio, and individual patient’s needs. Finally, “should” was used in recommendation for treatment discontinuation in case of severe adverse events or other life-threatening conditions, considering the importance of this issue.

“Could” was used in recommendations about patient’s compliance to treatment and follow-up, as, in this regard, it is particularly important to leave space for the physician to decide on a case by case basis. Although recommendations were taken from guidelines and clinical studies [17, 24], according to the group’s consensus reasons for missing infusions or follow-up should be evaluated individually, in the general context of disease and considering real patient’s will of adhering to treatment and follow-up. Also, no time deadline for patient’s compliance to follow-up assessments was included in statement, both because time for follow-up is different in different countries and because patients must be aware that all follow-up assessments are essential.

### Limitations

The study has limitations which require consideration. The consensus statement was focused mainly on initiation and follow up of enzyme replacement therapy for MPS IVA patients, not involving a comprehensive discussion on the whole management of disease, including criteria for diagnosis, supporting interventions and symptomatic treatments. Moreover, recommendations are based most exclusively on expert opinion, rather than on direct evidence collected in the centers. Therefore, despite the value of the statement, deriving from a widely shared consensus among experts on disease management and treatment, recommendations need to be implemented in real-world settings, to adjust them to country- or site-specific clinical practice and peculiar disease phenotypes in different patients.

## Conclusions

This consensus statement provides HCPs and Health Authorities involved in management and treatment of patients with Morquio A in Central and South-Eastern European countries with a set of guidance for optimal patient care. The main aim was to discuss European guidelines and best evidence in the context of real-life clinical practice in Central and South-Eastern Europe, to provide recommendations that could be suitable for all countries in the region. Importantly, as Morquio A is a rare disease, consideration should be given to the unique challenges of rare diseases, which are often underserviced, incur relatively high costs and may not be captured by conventional cost–benefit analyses. Also, important concern should be given to differences in criteria for patients’ access to treatment, that vary geographically due to different rules of the national health authorities and reimbursement schemes. In this scenario, it is important that inclusion/exclusion criteria for treatment are not arbitrary and do not discriminate any patient according to age or disease phenotype. In particular, these criteria should include, upon physician’s decision, patients at a late stage of disease or slowly progressing patients, where disease evolution cannot often be confirmed over a short period of time.

## Data Availability

Data sharing not applicable to this article as no datasets were produced or analyzed during the study.

## Abbreviations

3MSCT: Three-minute stair climb test
6MWT: Six-minutes walk test
ADAs: Anti-drug antibodies
ADL: Activities of daily living
AEs: Adverse events
C6S: Chondroitin-6-sulfate
CT scan: Computerized tomography scan
ECG: Electrocardiogram
echo: Echocardiogram
EMA: European Medicines Agency
EQ-5D-5L: 5-Level European Quality of Life-5 dimensions questionnaire
ERT: Enzyme replacement therapy
FEV1: Forced expiratory volume in 1 s
FVC: Forced vital capacity
GAG: Glycosaminoglycans
GALNS: N-acetylgalactosamine-6-sulfate sulfatase
HSCT: Hematopoietic stem cell transplantation
KS: Keratan sulphate
LSD: Lysosomal storage disorder
LVEF: Left ventricular ejection fraction
MPS: Mucopolysaccharidoses
MPS-HAQ: MPS health assessment questionnaire
MPSIVA: Mucopolysaccharidoses IVA
MRI: Magnetic resonance imaging
MVV: Maximum voluntary ventilation
PRO: Patient reported outcomes
SC: Steering Committee
T25FW: Timed 25-foot (7.6 m) walk
